# Biological and Physiochemical Methods of Biofilm Adhesion Resistance Control of Medical-Context Surface

**DOI:** 10.7150/ijbs.59025

**Published:** 2021-04-23

**Authors:** Yuanzhe Li, Xiang Li, Yu Hao, Yang Liu, ZhiLi Dong, Kexin Li

**Affiliations:** 1 School of Materials Science & Engineering, Nanyang Technological University, Singapore, 639798, Singapore.; 2 School of Chemistry and Biomolecules Engineering, National University of Singapore, Singapore, 637551, Singapore.; 3 School of Mechanical Engineering, Xiangtan University, Xiangtan, 411105, China.; 4 Hwa Chong International School, Singapore, 269783, Singapore.

**Keywords:** Biofilms adhesion, Medical-context surface, Biological methods, Physicochemical methods, Resistance control.

## Abstract

The formation of biofilms on medical-context surfaces gives the EPS embedded bacterial community protection and additional advantages that planktonic cells would not have such as increased antibiotic resistance and horizontal gene transfer. Bacterial cells tend to attach to a conditioning layer after overcoming possible electrical barriers and go through two phases of attachments: reversible and irreversible. In the first, bacterial attachment to the surface is reversible and occurs quickly whilst the latter is permanent and takes place over a longer period of time. Upon reaching a certain density in the bacterial community, quorum sensing causes phenotypical changes leading to a loss in motility and the production of EPS. This position paper seeks to address the problem of bacterial adhesion and biofilm formation for the medical surfaces by comparing inhabiting physicochemical interactions and biological mechanisms. Several physiochemical methodologies (e.g. ultrasonication, alternating magnetic field and chemical surface coating) and utilizing biological mechanisms (e.g. quorum quenching and EPS degrading enzymes) were suggested. The possible strategical applications of each category were suggested and evaluated to a balanced position to possibly eliminate the adhesion and formation of biofilms on medical-context surfaces.

## 1. Introduction

Biofilms are an aggregate of interface associated sessile bacteria embedded within a matrix of extracellular polymeric substances (EPS) 1-2. The multicellular community of bacteria encapsulated by EPS could either be made up of homogenous or heterogeneous populations 3-5. The biofilms go through a cycle of attachment of planktonic cells to an interface, micro-colony formation, biofilm maturation and secretion of biofilms and followed by the detachment (through different mechanism such as erosion, sloughing and dispersion) 6.

It is also important to note that biofilms could be found in an extensive range of environments including medical instruments, food processers, dental water lines, teeth, and water membrane surfaces 7. This is significant because biofilms can have both beneficial and unfavorable impacts on our daily lives. Some of the benefits of biofilms are the removal of contaminants e.g. ammonia and oil spills and purification of industrial wastewater 8. On the other hand, the negative impacts of biofilm growth include an increased cleaning and maintenance cost in a myriad number of industries, food and water contamination and an increased bacterial resistance to the human immune system 9.

Medical biofilms are ubiquitous in the clinical environment and it is also the main problem that all the surgeries are facing. The main reason is that microorganisms may adhere to the surface of medical devices and form biofilms, and recurrence is very common in these medical devices. Compared with their floating states, they show different growth rates, structural characteristics, and protective mechanisms against drugs and host immunity. A better understanding of the relevant regulatory factors, survival status, and drug resistance mechanisms of biofilm formation can enable more effective development of innovative strategies for the diagnosis of biofilm-related infections, as well as new methods for the treatment and prevention of medical device-related infections. E.g. one way of getting rid of this problem is the application of biocides to clear the biofilms. However, some biocides may be toxic to non-specific organisms and pollute the medical environment 10-13. Chlorine for example, produces harmful disinfectant by-products when it is exposed to high levels of organic matter. Furthermore, biofilms generally increase the resistance of the sessile bacterial community which renders the biocide ineffective or requires continuous addition of higher concentrations of microbicides 14-16. All these are neither desirable nor economical in the long run.

To address this problem, this paper is more focusing on the control of the microbial adhesion on medical context surfaces without the use of microbicides. The following areas will be covered: the microbial adhesion and biofilm formation, biological and physiochemical mechanisms on the resistance of biofilms and the new opportunities of antibiofouling strategies for medical-context surface.

## 2. Occurrence of biofilm attachment

### 2.1. Pre-attachment process

#### 2.1.1. Conditioning Layer

The surface of the substratum plays an important role in the adhesion of micro-organisms 12. One of the key developments before the adhesion of bacteria to a surface is the formation of the conditioning layer. The conditioning layer is composed of cellular components, organic macromolecules and inorganic compounds 8-9. The conditioning layer also encourages the adhesion and concentration of bacteria, leading to biofilm growth due to the increase in nutrient concentration on the surface.

#### 2.1.2. Biofilm Motility

The biofilm attachment to a surface is highly dependent on its motile ability or the fluid velocity. It tends to move across the bulk liquid and surfaces either through *motile appendages* (e.g. *flagella* and *pili*) or if lacking motile appendages, Brownian motion (random, uncontrolled movement of particles in a fluid) 10. For the former, it is able to adhere to surface regardless of flow velocities and latter are only able to adhere at low and medium fluid velocities 13,15,17.

### 2.2. Attachment process

#### 2.2.1. DVLO Theory

As bacteria sense a change in pH, osmolality and flagella rotation due to its proximity to a surface, it begins the process of attachment 13. The process of attachment, however, is highly affected by the surface attractive and repulsive energy as described by the DLVO theory 14. The DVLO theory, a summation of van der Waals and coulomb interactions, defines the net interaction between a cell and a surface as the sum of attractive van der Waals forces and repulsive electrostatic interactions 8,15. Van der Waals is the dominant attractive force in the vicinity of a surface where cells are unable to separate from the surface by Brownian motion; Coulomb interaction on the other hand is the dominant force at a distance away from the surface due to a decrease in van der Waals forces 15.

Van der Waals interactions are usually weak, noncovalent attractive forces 16. The repulsive forces by Coulomb interaction on the other hand are due to the formation of an electrical double layer on both the cell and surface 8. The ionic strength and pH of the bulk liquid is capable of influencing the charge of a cell wall and the substratum due to counter ions being attracted to charged particles in an aqueous solution 14. These counter ions attracted by both bacteria and surface in an aqueous solution generally cause an overlap of negatively changed electrical double layer that repulses the bacterial cell from the surface. These attractive (van der Waals) and repulsive (Coulomb interaction) forces will facilitate or prevent the attachment of bacteria cell to the surface of a material. However, it has been observed that one of the ways that bacteria are able to overcome the repulsive Coulomb forces is through the use of long stranded eDNA that penetrates the negative electrical barrier, thus aiding the attachment of a bacterial cell to a surface 11,17.

#### 2.2.2. Reversible Attachment

Bacterial attachment to a surface can be split into two phases: reversible attachment and irreversible attachment 13. Reversible attachment occurs rapidly and begins once primary contact with a surface is made 9,18. It is transient and reversible during this short period and bacteria are able to detach and revert back to planktonic form 2,19. During reversible attachment, bacteria cells either uses nanofibers (e.g. pili and flagella) with the presence of adhesins or produces EPS to bridge between the cell body and substratum and increase adhesive forces 20. It is noted that whilst EPS is paramount for biofilm development, it is also playing an essential role in the initial attachment of the bacteria and functions as an adhesion. Besides the fore-mentioned eDNA, bacterial nanofibers and EPS, other adhesins include proteins and non-proteinaceous adhesive polysaccharide capsules 19.

#### 2.2.3. Irreversible Attachment

After reversible attachment, irreversible adhesion occurs slowly to the surface 13. Irreversible attachment is characterized by stronger induced cell-surface interactions and smaller cell-surface distance (less than 1.5nm) 14,21. This allows adhesins (ligands) that are exposed on the cell surface to form a “key-lock” bond between the cell and the substratum. An alteration of gene expression will also begin which pivots the cellular processes towards biofilm formation. This is due to mechano-sensing where the signal transduction pathway senses and responds to forces produced by the surface attachment. Mechano-sensing also generates orbiting motility which decreases the range of the bacteria's motility and increases the period spent near the region of the surface 9,22-24. It is also suggested that during irreversible attachment, bacteria would have overcome the repulsive forces of the electrical double layer in the reversible attachment stage and begin chemical reactions (e.g. oxidation and hydration) upon contact with the bulk lattice of the conditioning layer 8,25.

### 2.3. Biofilm growth

#### 2.3.1. Quorum Sensing

Once a certain concentration/density of bacteria is reached, quorum sensing (QS) mechanisms activates the genetic mechanisms to induce changes in bacterial gene transcriptions 18,19. There are two different types of quorum chemical signalling molecules produced by bacteria. Gram-negative bacteria produce acyl homoserine lactone (AHL) and gram-positive bacteria produces autoinducer peptides (AIP) 20,26.

#### 2.3.2. EPS Production

The coordinated bacteria gene transcriptional changes through the QS system catalyzes secondary signalling molecules (such as c-di-GMP) which downregulates motile appendages such as flagella and promotes EPS production 6,27. The continuous EPS production by the bacteria community will enhance the integrity of the biofilm structures 28.

## 3. Biological methods of biofilm adhesion resistance control

**Figure 1:**
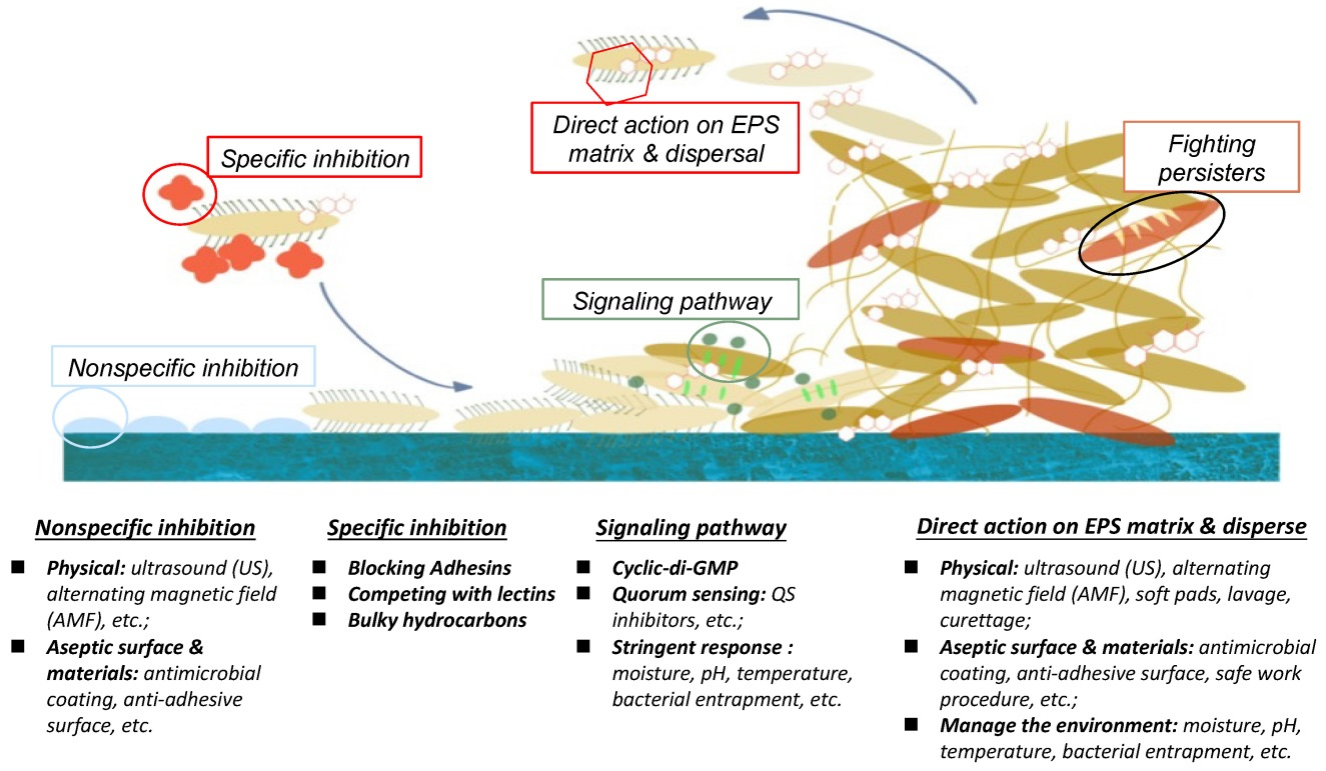
Different inhibition methodologies, signal pathway activities, and EPS actions for biofilm-growth control.

There are numerous biological and physicochemical mechanisms to inhibit or eliminate biofilm formation on a surface. As summarized in Fig. 1, these include non-specific inhibition (e.g. physical methodology, aseptic surface materials), specific inhibition (e.g. blocking adhesins, competing with lectins), disrupting signalling pathways, and enzymatic action on EPS matrix and destruction of persisters 19,29. This position paper will be mainly focusing on quorum quenching and enzymatic action.

### 3.1. EPS degradation enzymes

The EPS secreted by bacteria offers protection from the surrounding environment and gives the embedded sessile bacterial community certain advantages that planktonic bacteria do not have 6. By removing the EPS matrix, the bacterial community are without the EPS protection would decrease due to a lowering of fluid frictional resistance. EPS degrading enzymes are able to breakdown the EPS matrix, leading to a reduction of cell-to-cell and cell-to-surface associations during attachment, removal of biofilm colonies, increased microbial cells sensitivity to antibiotics, decreased tolerance to environmental factors and QS disruption 6,19,30. Though there are many applications and benefits to the use of enzymes, three particular disadvantages that should be highlighted is its high cost in operation, restricted spectrum of action per type of enzyme and short enzymatic lifetime 17,31. The source of enzymes can come through bacteriophages or synthetic biological engineering.

One of the main applications of enzymes during the first 24 hours of microbial attachment is the inhibition and degradation of the EPS matrix for its reversible attachment process. Different enzymes such as glycosidases, proteases and DNase degrade different components in the EPS (specifically polysaccharides, proteins and eDNA respectively) 6,32-33. This destabilizes the biofilm by altering the biofilm architecture and increasing the penetration of antibiotics 34. The degradation of eDNA by DNase 1 is through the cleavage of phosphodiester bonds of single stranded and double stranded DNA. Examples of each type of enzymes are: Glycosidases (α-amylase, β-glucanase & DspB), Proteinase (K & Trypsin) and DNase (DNase 1) 34. A list of different types of DNase from bacteria and its effect on biofilm formation is found in Table 1.

An example of how enzyme can be applied conventionally is the use of enzyme detergents which have the advantages of being sensitive to materials that can be harmed by harsh chemicals or high temperatures. By using enzyme detergents that contain a mixture of protease, D-Nase I, amylase and cellulose, it can destabilize the biofilm EPS and improve the efficiency of biofilm removal 22,36.

### 3.2. Quorum quenching

Quorum sensing (QS) is a cell-to-cell communication system that controls gene expression in a cell density dependent manner 19,35. QS regulates a diverse range of phenotypic expressions including virulence, motility, luminescence and biofilm formation 36. QS signalling molecules, used to mediate bacterial ability to sense the surrounding cell density, have these particular characteristics: (i) production happens in specific growth stages or a feedback to certain environmental challenges; (ii) accumulate in the surrounding environment and be recognized by bacterial receptors; (iii) an accumulation of a threshold QS signal concentration results in physiological response; and (iv) results in phenotypic changes. Even in bacteria such as *Escherichia coli* and *Salmonella* that do not produce AHLs (gram negative QS signalling molecule), it still possesses AHL response regulator protein, enabling it to respond to AHLs made by other bacteria. A third QS signalling molecule: Autoinducer-2 (AI-2), has been proposed as the signalling molecule used on an intra-species and inter-species level; facilitating communications between gram positive and negative bacteria 37-39. It is important to note that although QS is essential for the formation of biofilms, the loss of this cell to cell communication system does not cause a lethal effect on bacterial cells 20. And hence, the disruption of the QS pathway (quorum quenching) is able to cause microbial control and not have a biocidal effect on bacterial cells.

**Figure 2:**
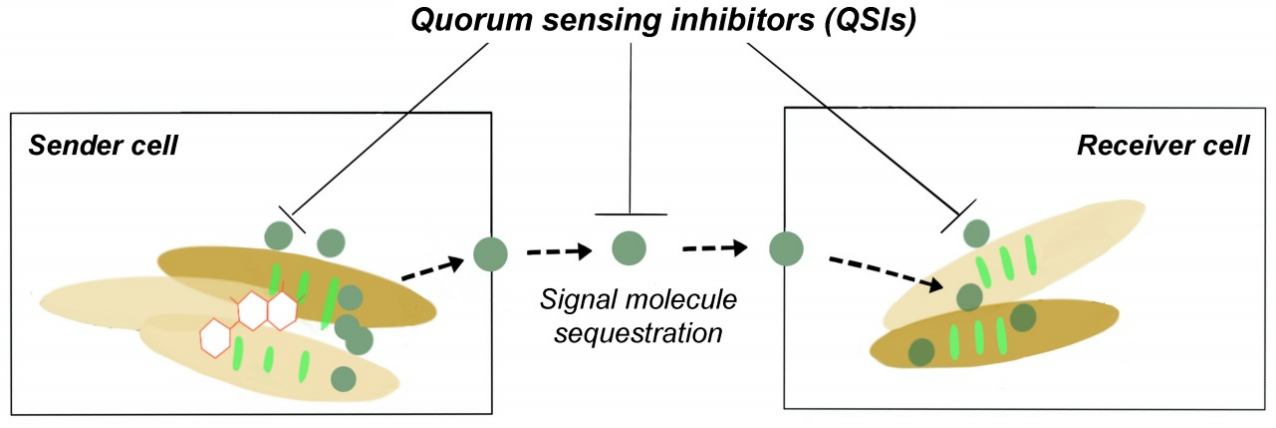
Schematic diagram of QSI prevents biofilm formation.

Quorum quenching as shown in Fig. 2, is the process of disrupting QS communications through any of these three different mechanisms: (i) QS signalling molecule production inhibition, (ii) QS signalling molecules inactivation, and (iii) QS signal molecule reception disruption 20,23,40. QS quenching is able disrupt the establishment and maintenance of the production of the EPS matrix, leading to a loss in biofilm structural integrity and increased bacterial susceptibility to antibiotics. For example, there are various natural and synthetic QS inhibiting (QSI) compounds that disrupt QS pathways including as vanillin (VA) and cinnamaldehyde (CNMA) 41-42. One of the mechanisms how QSIs work is by hydrolyzing the lactone bond of a QS molecules such as AHLs thus inactivating the signalling molecule itself 6,43. VA, a non-toxic food additive, was noted to be able to suppress EPS secretion and inhibit both homogenous and heterogeneous bacterial biofilm communities by 69.8 %. This was done without any significant change to the live cell biomass 24,35. A list of different QSIs and mode of actions can be found in Table 2.

A particular number of QSIs found in literature use enzymes for quorum quenching. Examples of these are acylase 1 (degrade the QS signalling molecule by cleaving the AHL bond), lactonase (targets the homoserine lactone ring), decarboxylase and demainse 15,43. Some of these QSI enzymes are produced by bacteria. The Bacillus species for example produce enzymes that block QS communications in gram-negative bacteria that are based on homoserine lactone signalling molecules 44. Another study showed that biofilm formation by *Pseudomonas Putida* was greatly reduced by the addition of acylase concentration (21mg/L to control biofouling in a bench-scale crossflow) 45. Hence, there are a wide array of quorum quenching enzymes available to inhibit the growth of early biofilms 46-47.

The two biological mechanisms strategies - quorum quenching and enzymatic matrix degradation, can be used to inhibit the growth of biofilms on the medical context. However, the application cannot simply be pouring QSIs and enzyme concentrate onto the surface as it would be economically wasteful and possibly ineffective due to the surrounding water flow. Besides, horizontal gene transfer across the biofilm results in increased antibiotic resistance. Some bacterial cells also enter a dormant mode where they become persister cells and are able to resist antibiotics. One method of utilizing these two biological mechanisms is through surface coating of QSIs and EPS degrading enzyme by immobilizing QSIs and enzymes on the surface 48. Other possible methods magnetic immobilized enzyme (e.g. acylase) carriers (MECs) 35. Surface coating of QSIs and enzymes are noted to be able to reduce the formation of biofilms.

In addition, the usage of the right enzyme such as benzonase could potentially have remarkable outcome due to its wide range of specificity - degrading all forms of DNA and RNA and its ability able to work over a broad range of pH and temperatures. As enzymes can have a short life span, immobilization could also be advantageous as it causes most enzymes to become more efficient and stable 17,36. Kim JH showed that by immobilizing acylase on a surface, it was able to prohibit biofilm formation and maintain more than 90% enzymatic activity over an extended 20 reaction and washing cycles in nano-filtration 37.

## 4. Physical methods of biofilm adhesion resistance control

Physical biofilm-adhesion-resistance-control methods, which include flushing, swabbing, air scouring, pigging, and ultrasonic scaling, have been existing for the effectively removal of biofilms. This position paper selects two typical methods, ultrasonic and alternating magnetic field, to explain the mechanism and the efficiency of physical methods of biofilm adhesion resistance control in the medical context.

### 4.1. Ultrasonic (US) removal

**Figure 3:**
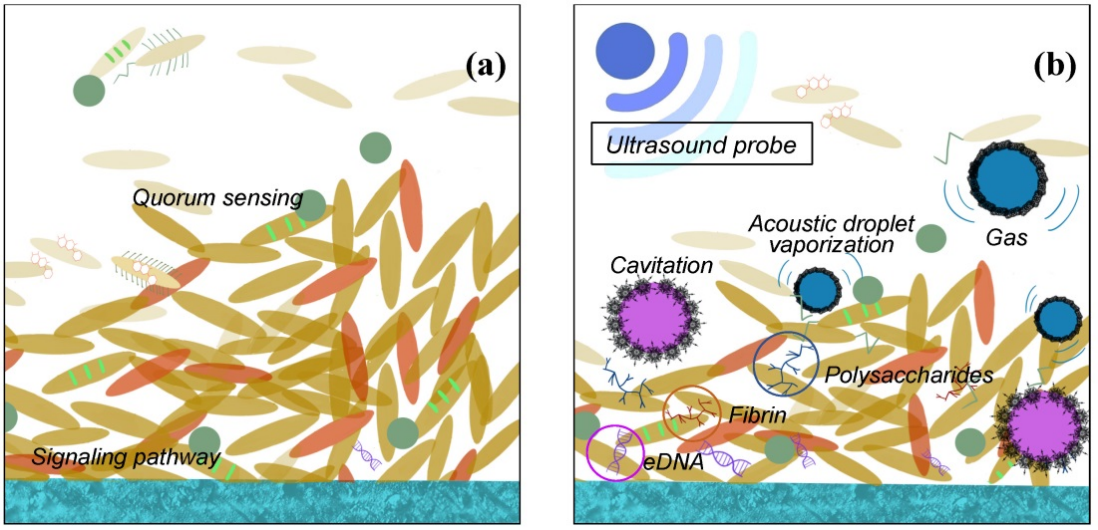
US biological effects on biofilm cell (a) before and (b) after ultrasound probe is applied.

It is well known that ultrasonic could be used to clean surfaces with complicated spatial structure. The ultrasonic field contains high energy and is able to conduct reflection on the surface and go through different cavitation with porous structure. It is believed that the shear forces in the acoustic droplet vaporization are also responsible for the detachment of the bacteria as indicated in Fig. 3. And under high frequency and intensity of US, the eDNA, fibrin, and polysaccharides may be leaking to the environment after the bacteria membrane is damaged. Actually, the ultrasonic activity can end up with two ways of bacterial effect: a bactericidal effect, or a stimulation of growth 49-50.

The most important effect of bactericidal activity is considered to be the transient cavitation, which occurs at high intensity and low frequencies. High intensity is linked to the destruction of the EPS matrix with a bactericidal effect. However, low intensity appears to stimulate the growth of the bacteria because of the destroying the structures of the EPS matrix and out layer of the biofilm clusters only. The increasing transport of nutrient and oxygen in the deeper layers enable the biofilm to develop again 49,51. Low frequency might be easier to conduct diffraction and able to avoid the obstacle, while high frequency, though with higher energy, the smaller wavelength may not let the microwave drive around the block inside the biofilm, so the lower frequency is preferred. Moreover, the generated acoustic droplet vaporization to accelerate the fluid around them violently. When these bubbles vaporise and collapse, the adiabatic heating of the internal gas will generate high temperatures and provide free radicals and high fluid shear force. After certain numbers of cavitation collapse, the cell membranes may be destroyed by high shear rates or damaged by the heat or radicals 50-52. Furthermore, the cavitation adjacent to high-density surfaces can generate even higher stress on cell membrane during the process of the bubble expansion than the low-density ones. The other ways, such as US field increasing chemical disinfectant activity and producing a mechanical oscillation of a tip are also reasonable in the explanation of the bactericidal response with certain evidence to support.

**Figure 4:**
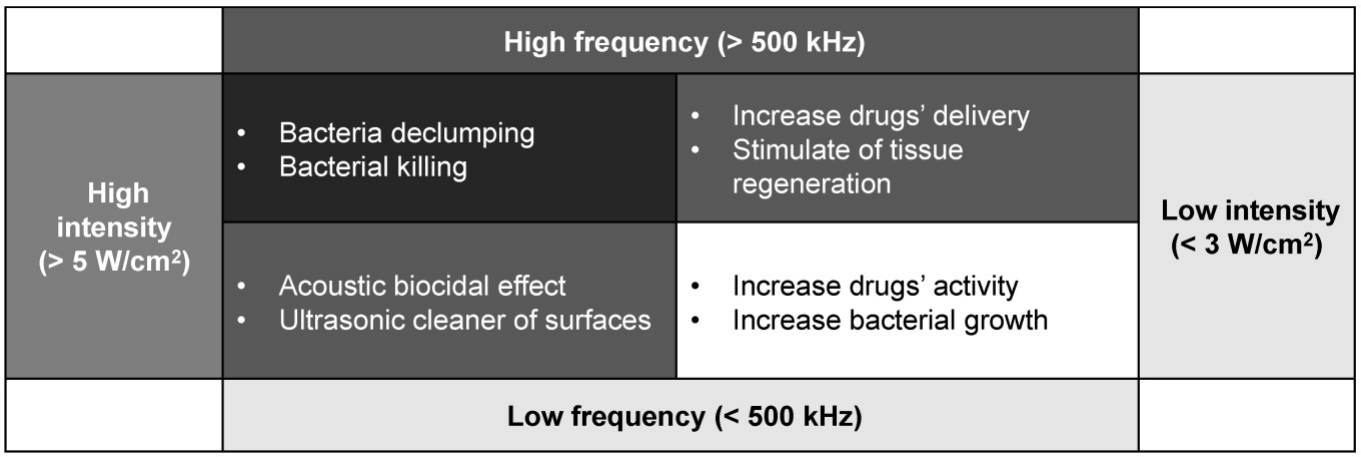
US biological trends depending on different frequency and intensity levels.

To sum up, when bacteria are exposed to the US field, both destruction and stimulation effects will occur. The US biological effect is strongly influenced by the effect of ultrasonic cavitation, especially intensity and frequency ultrasonic, or to the contact of the transducer with the biofilm 51,53-54. The response of the biofilm to the ultrasonic depends on various factors. Generally, the response is controlled by the intensity and frequency as the Fig. 4 concluded above.

### 4.2. Alternating magnetic field (AMF) removal

The feasibility and safety of using alternating magnetic fields (AMF) exposures to eradicate biofilm on metal surfaces has been proved, and magnetic iron oxide and similar nanoparticles has been increasingly used in various biomedical technologies in recent research 55-56. Introducing a non-invasive method for thermal destruction of biofilm on metallic implants with high-frequency (>100 kHz) alternating magnetic fields (AMF) becomes a solution for the biofilm removal especially in the treatment of prosthetic joint infection and the surgical cleaning of infected joint 57-58.

**Figure 5:**
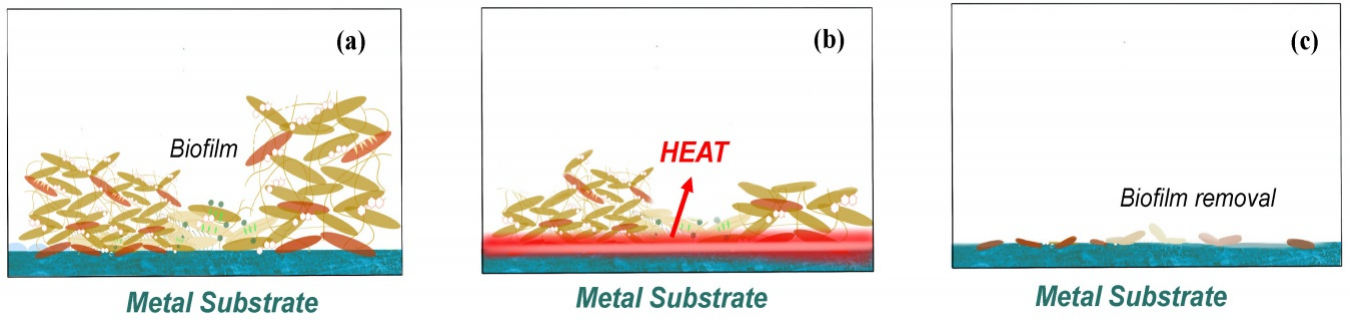
Biological effects of how biofilm on the implant surface removed by AMF (a) before AMF is applied, (b) heat generate with AMF process, and (c) biofilm removal by AMF.

The reason why heat is generating is due to the induction of electromagnetism in metals or a conductor as indicated in Fig. 5. This current is also known as Foucault current 59. As the conductor moving in a non-uniform magnetic field, or a conductor is stationary but has a magnetic field that varies with time, or both situations occur at the same time, it results in relative cutting of the magnetic force line by the conductor. According to the law of electromagnetic induction, the induction electromotive force is generated in the conductor, thus driving current. The distribution of the current in the conductor varies with the surface shape of the conductor and the distribution of the magnetic field. A vortex will produce heat in conductor 60-62.

Plenty of recent researches are discuss on the removal efficiency by the alternating magnetic field (AMF) removal, and some of the authors argued that it should be more accurately described as rotating magnetic field (RMF) rather than AMF, as AMF refers to the induction heating, which is the heating metal under high frequency changes but with random shaped electromagnetic field, while the rotating magnetic field (RMF) refers to the design with practical application in the induction coil, which can generate strong electromagnetic induction from forming on the surface of eddy current, though rely on the internal resistance of material itself, rapid heating even shorter 63-65. So, discussion the only focusing on the relationship between time and removal efficiency is meaningless. But according to the matter of washer surface change, it is not hard to conclude 85 °C might be such a critical temperature to realize the killing of most bacteria or help to conduct the biofilm removal. Similar conclusions of critical temperature for most bacteria could also be found in other AMF/RMF related essays 66. And such AMF methods may act at any steps of the biofilm attachment process with no obvious biofilm resistance.

The reason why this article does not include the discussion of other physical methods, such as flushing, swabbing and pigging, is due to the advantages that magnetic field and ultrasonic have the over other physical methods are: (i) no need direct contact with the surface and realize the bactericidal effect; (ii) able to provide the availability to remove the biofilm on complex structure as well as giant surface or space; (iii) they have a relatively high removal efficiency, especially for the first 24 hrs biofilm adhesion, within a short period of time and such bactericidal effect may also influence the quorum sensing, as in the following 1 to 2 hrs the adhesion rate keeps relative low 67.

## 5. Chemical methods of biofilm adhesion resistance control

There are mainly four chemical biofilm-adhesion-resistance-control methods that commonly used in cleaning the clinical settings and medical tool and equipment. These methods include detergent, hydrogen peroxide washing, bactericidal/bacteriostatic paint, and anti-adhesion coating 68. This chapter is to briefly review the bactericidal/bacteriostatic paint and anti-adhesion coating after considering its economy, feasibility and its promising application in the area of medical surface.

### 5.1. Contact bactericidal/bacteriostatic paint

Contact antimicrobial paints are one of the most common antimicrobial paints that have been studied. Organic or inorganic biocides with strong antimicrobial properties are usually immobilised on the surface of biomedical materials by physical adsorption or chemical bonding, and bacteria are rapidly killed by direct contact with the paint. Among them, quaternary ammonium salts (QAS) are the most used organic bactericides 69-73, which will attract bacteria through electrostatic interactions first, facilitate the hydrophobic alkyl chains of QAS to pierce the cell wall of bacteria attached to the surface of the material, and then dissolve the cell membrane and destabilize the intracellular matrix through the contact mechanism 74.

As early as 1916, Jacobs et al. found that most of the QAS obtained by the reaction of hexamethylenetetramine with various substituted benzyl or alkyl halides had strong bactericidal activity 75. Guo et al. 76 pre-treated glass fibre membranes with plasma bombardment and further used chemical grafting to anchor QAS molecules on the surface of glass fibre membranes and found that the antibacterial effect on the surface of glass fibre membranes was significant after modification by QAS 77-78. Wan et al. 79 synthesised QAS copolymers, which exhibited excellent antibacterial properties due to their better water solubility and easier access to bacteria. In addition, Lv et al. 80 further investigated the antibacterial activity of QAS polymers with different alkyl chain lengths and found that the chain length had a significant effect on the antibacterial performance of QAS, with the longer the chain the lower the antibacterial activity. The increase in chain length increases the tendency of QAS to aggregate and form spheres, making the polymer inaccessible to bacterial cells and weakening its antibacterial activity. Recently, Li et al. 81 designed specific structures of QAS with maleic pine acid and used them for surface modification of cotton textile dressings. It was found that the modified cotton spun dressings not only had strong antimicrobial broad-spectrum and durability, but also excellent biocompatibility, which is promising for medical wound dressing applications. In addition, antimicrobial peptides 82-84 are also a class of organic antimicrobial agents that have been widely studied and applied, with the advantages of broad antimicrobial spectrum, high antibacterial and anti-endotoxic activity, target specificity and low drug resistance 85. It is believed that the bactericidal mechanism of antimicrobial peptides is based on electrostatic attraction to the surface of bacterial membranes, which disrupts cell integrity and produces perforation, resulting in the spillage of bacterial contents and death 86. Eby et al. 87 used cationic antimicrobial peptides to modulate the *in situ* biomineralization of silica (SiO_2_) and titanium dioxide (TiO_2_) to form SiO_2_, TiO_2_ and antimicrobial peptide composite antimicrobial nanoparticles, which not only maintained the excellent antimicrobial properties of the antimicrobial peptides, but also protected the antimicrobial peptides from protease catalytic degradation and promoted the sustained release of the antimicrobial components. Yazici et al. 88 designed a bifunctional chimeric antimicrobial peptide with a special structure. One end of the chimeric peptide is easily adhered to the surface of commonly used titanium implants, while the exposed peptide molecule at the other end is effective against invading bacteria, especially Streptococcus pyogenes, Staphylococcus epidermidis and Escherichia coli. Kazemzadeh-Narbat et al 89 further constructed a multilayer composite antimicrobial paint with slow-release antimicrobial peptides on the surface of titanium alloy. The paint consisted of vertically oriented TiO_2_ nanotube layer, ultrathin calcium phosphate layer and phospholipid film infiltrated antimicrobial peptide layer, which not only had excellent antimicrobial activity, but also was non-cytotoxic to osteoblasts and had good hemocompatibility. In addition, the antimicrobial peptide can be directly deposited on the surface of the catheter, and the surface-modified antimicrobial catheter maintains a long-lasting antimicrobial activity for 21 days with no toxicity to humans 90. Recently, Acosta et al. 91 used biotechnology to obtain recombinant protein polymers to directly immobilise antimicrobial peptides on the surface of titanium alloys. The antimicrobial paint was found to exhibit excellent antimicrobial activity under dynamic biofilm incubation conditions, with good cytocompatibility and low toxicity to mammalian cells, which is a good medical advantage in the prevention of implant infections.

### 5.2. Anti-adhesion coating

**Figure 6:**
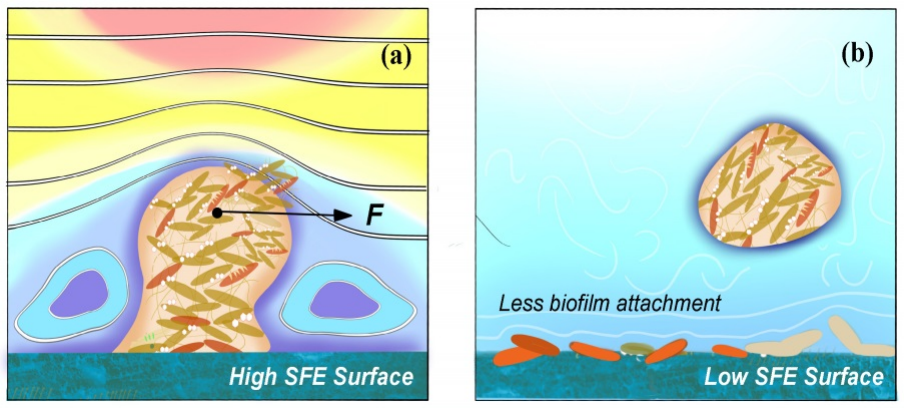
Schematic diagrams of how (a) high SFE and (b) low SFE coating surface influence the biofilm formation.

Anti-adhesion coating is able to prevent biofilm from adhering on the surface at early stage, which should be desirable in all kinds of medical settings. The anti-adhesion coating surface generally needs considerations for four characteristics, which includes (i) chemical composition and reactivity, (ii) hydrophilicity and hydrophobicity, (iii) surface texture, and (iv) low surface energy (SFE) as shown in Fig. 6 92.

Strategies for the construction of anti-adhesive and anti-bacterial coatings are mainly based on super-hydrophilic or super-hydrophobic anti-adhesive functional surfaces 93. On the one hand, hydrophilic polymers are directly used to modify the surface of the materials or directly prepare high water content hydrogel coatings, these polymer brushes or hydrogel coatings can have a strong binding effect on water molecules through hydrogen bonding, electrostatic and van der Waals force interactions to form a hydrated layer, making it necessary for proteins or cells to overcome these physical barriers and energy barriers in the process of adhesion to the material surface, thus hindering protein adhesion and on the other hand, fluoropolymers and increased roughness have been used to modify the surface of materials with super hydrophobicity 94-95. Superhydrophobic surfaces are effective in inhibiting the adhesion of biomolecules such as proteins and bacteria due to their extremely low surface energy 96. Both strategies do not use antimicrobial agents or antibacterial drugs, and the antibacterial process is safe as no heavy metals or antibiotics are released, which can cause side effects and contamination during the use of medical materials. However, anti-adhesive antibacterial coatings are often only initially antibacterial and are not effective in complex environments with large and diverse biomolecules, such as *in vivo*, and are not sustainable 97. Therefore, it is often necessary to combine other antimicrobial technologies, such as anti-adhesive and bactericidal technologies, to create an ideal antimicrobial surface with synergistic anti-adhesive and bactericidal effects.

The recent research of modified antibiofouling polyurea coating reveals that the modified polyurea anti-biofouling coating indicates hydrophobic characteristic; the nano-titanium dioxide may generate reactive oxygen species to kill the bacteria; and the riblet surface textures formed by nano-titanium dioxide and even nano-zinc oxide are capable to perform anti-bacteria characteristics as well 98-99. Not using the other chemical agent such as silver and organic solvent, is because the toxic effects could be observed at very low concentrations, bioaccumulation (removal capacity exceeded) and biomagnification (accumulation up the food chain/web) may also induce more toxic substance into organisms and environment. And the combination for physical method, ultrasonic and alternating field, and chemical method, building a chemical inert, low energy and smooth coating, could be a series solution in the biofilm removal in medical depends on the different stages of the biofilm 100.

## 6. Novel Strategies of Biofilm-Resistance Control for Medical Context

The widespread use of biomedical materials in clinical treatment has led to an increasing number of infections, which pose a serious threat to life and health. The use of suitable surface modifications to construct antimicrobial paints on the surface of biomedical materials is an effective way to address these infections of medical origin. Currently, the main types of antimicrobial paints are contact antimicrobial paints, anti-adhesive antimicrobial/sterilisation paints and smart antimicrobial paints. Among them, smart antimicrobial paints can not only solve the problem of bacterial cadaver adhesion and agglomeration in contact antimicrobial paints, but also achieve controlled release of antimicrobial substances through physical and chemical excitation response mechanisms to avoid environmental hazards; and often achieve efficient antimicrobial efficacy through the synergistic effect of different antimicrobial methods, which is an important direction for the future development of antimicrobial paints.

Through the discussion above about the character and the mechanism that might be used in daily life, so here just list three of the innovative physicochemical routes that come up with, based on that matter of the facts, to remove the biofilm. And these physicochemical routes are especially designed for the specific use in medical context surfaces. (i)** heat resisting and anti-adhesion coating with metal substrate,** i.e. the heating resisting coating is designed to undertake the high temperature which is generated by the alternating magnetic field around metal substrate. Hence, the biofilm will automatically remove and separate from the coating and the coating will still stick on the metal substrate. Besides, the super hydrophobic property is used to reduce the further attachment and adhesion of the biofilm due to its low surface energy. (ii)** scratch proof and anti-adhesion coating, i.e.** the scratch proof coating is made for the ultrasonic cleaning and brush cleaning to remove the biofilm. The ultrasonic is used for the early stage of the biofilm adhesion while the brush cleaning using some detergent or enzyme. The super hydrophobic property is also used to reduce the further attachment and adhesion of the biofilm. (iii) **heat resisting + scratch proof with metal substrate,** i.e. combination of heat resisting, and scratch proof properties enhance the stability and durability under cruel physical condition, such as high/low temperature, high pressure and even some chemical corrosion. Metal substrate continuously uses for the heat effect for the biofilm removal 101-102.

In practice, for biomedical materials implanted in the body, long-term contact with human tissues requires higher performance of the surface antimicrobial coating, which must not only have efficient and durable antimicrobial properties and stability, but also be non-toxic and non-hazardous, and have excellent biocompatibility. In addition, due to the variety and number of different types of implantable and *in vitro* medical auxiliary materials, the construction of antimicrobial coatings often requires different methods of surface modification depending on the physical and chemical properties of the actual material surface, which greatly increases the difficulty of industrial mass production of antimicrobial functional biomedical materials. Therefore, it is crucial to develop antimicrobial coatings with excellent broad-spectrum and efficient antimicrobial properties, non-toxic, non-polluting, non-resistant, durable and stable antimicrobial coatings as well as universal surface antimicrobial coating construction methods. It is believed that in the future, antimicrobial coatings will further enhance the antimicrobial properties and functionality of biomedical materials, minimising the risk of medical-derived infections and reducing medical costs for the benefit of mankind.

## 7. Conclusion

This paper reviews the current trend of the biological and physicochemical methods, which have been broadly used in the bacterial removal of different variations of medical context. The synergistic effect of anti-adhesion composition and antibacterial agent, including degradation QSIs, enzymes, and many other chemical detergents, are important ways to obtain high-efficiency antibacterial efficiency. Although the disadvantages of QSIs and enzymes are the eventual loss of enzymes, high cost, and the formation of a conditioning later, the construction of hydrophobic anti-bacterial coatings is still a promising anti functionality for medical applications, i.e. the hydrophobicity of the coating can effectively prevent moisture and dirt and reduce the growth of mold on the surface of the object, and the bacteria will not adhere to it due to the low surface energy.

## Figures and Tables

**Table 1:** Type of DNase treatment and mode of action against biofilm formation 25,34.

Bacterium	Type of DNase	Mode of action (Disruption of pre-existing biofilm/Prevention of biofilm formation)
**Gram-negative bacteria**		
*Acinetobacter baumannii*	DNase I	Disruption
*Actinobacillus actinomycetemcomitans*	DNase I	Disruption
*Bdellovibrio bacteriovorus*	DNase I	Disruption
*Bordetella pertussis*	DNase I	Disruption
*Bordetella bronchiseptica*	DNase I	Disruption
*Campylobacter jejuni*	DNase I	Disruption
*Comamonas denitficans*	DNase I	Prevention
*Escherichia coli (E. coli)*	DNase I, NucB	Disruption
*Haemophllus influenzae*	DNase I	Disruption & Prevention
*Klebsiella pneumoniae*	DNase I	Disruption
*Neisseria meningitides*	DNase I, Varidase	Prevention
*Pseudomonoas aeruginosa* *(P. aemginosa)*	DNase I L2	Disruption & Prevention
*Shewanella oneidensis*	DNase I	Disruption & Prevention
*Vibrio cholera*	DNase I, λExonuclease	Disruption
**Gram-positive bacteria**		
*Bacillus licheniformis*	NucB	Disruption
*Bacillus subtilis (B. subtilis)*	NucB	Disruption
*Enterococcus faecalis*	DNase I	Disruption
*Listeria monocytogenes*	DNase I	Disruption & Prevention
*Staphylococcus aureus (S. aureus)*	DNase I, rhDNase I, NucB	Disruption & Prevention
*Staphylococcus epidermidis* *(S. epidermidis)*	DNase I, DNase I L2, NucB	Disruption
*Staphylococcus haemolyticus*	DNase I	Disruption
*Streptococcus anginosus*	NucB	Disruption
*Streptococcus constellatus*	NucB	Disruption
*Streptococcus salivarius*	NucB	Disruption
*Staphylococcus lugdunesis*	NucB	Disruption
*Streptococcus intermedius*	DNase I, NucB	Disruption
*Streptococcus mutans*	DNase I	Disruption
*Streptococcus pneumonlae*	rhDNase I	Prevention
*Streptococcus pyogenes*	DNase I	Prevention

**Table 2:** Quorum sensing inhibitors (QSIs), QSI sources, and action mode 35.

QS inhibitor	Source of organism	Action mode
**Natural Inhibitors**		
AHL-lactonase	*Bacillus cereus* *Agrobacterium tumefaciens* *Halomonas sp. strain 33*	AHLs degradation
AHL-acylase	*Tenacibaculum discolor strain 20J* *Hyphamonas sp. DG895*	AHLs degradation C4HSL and 30C12-HSL
AHL-oxidase	*Bacillus megaterium*	C4HSL and 30C12HSL
AHL-oxidoreductase	*Burkholderia strain GG4*	30C6HSL
Lactones	*Streptomycetes spp.*	Mimic AHL signals
Halogenated Furanones	*Delisea pulchra*	Mimic AHL signals and inhibit gene expression
Vanillin (4-Hydroxy-3-methoxybenzaldehyde)	Vanilla beans extract (Vanilla *planifolia Andrews*)	Interfere with AHL receptors. Inhibit C4-HSI C6-HSL, C8-HSL, 3-oxo-C8-HSL
Ajoene (1-Allyldisulfanyl-3-(prop-2-ene-1sulfinyl)-propene)	Garlic extract (*Allium sativum*)	Blocks the QS-regulated productions of rhamnolipid resulting in phagocytosis of biofilm. Targets Gac/RSM part of QS and lowers the expression of regulatory RNAs in *P. aeruginosa* PAO1
Iberin (1-Isothiocyanato-3-(methylsulfinyl) propane)	Horseradish extract (*Armoracia rusticana*)	Inhibit expression of QS-regulated lasB-gfp and rhlA-gfp genes responsible for virulence factor in *P. aeruginosa*
*Piper betle*	*Piper betle* extract	Inhibit QS-mediated biofilm formation in P. aeruginosa
*Garlic*	Garlic extract	Interferes with expression of QS-controlled virulence genes in *P. aeruginosa*
Tumonoic acids	Blennothrix cantharidosmum	Compete with QS signals
Curcumin	Turmeric	Reduction of AHL production
**Synthetic inhibitors**		
2-aminophenol	Synthetic	QS gene expression inhibitor
Triclosan	Synthetic	Inhibitor of the enoyl-ACP reductase
Furaly hydrazide	Synthetic	Mimic AHL signals
Furanone F3 and F4	Synthetic	Reduce 3OC12HSL dependent QscR activity
Blastmycinolactol (Lactone)	Synthetic	Mimic AHL signals

**Table 3:** Biological effects on different US frequency and intensity 51-52.

Frequency	Intensity	Bacterium	Effect of ultrasonic
20 kHz	0.2 ~ 2 W/cm^2^	*B. subtilis*	Competition of two bacterial responses: bacterial killing and bacterial declumping.
26 kHz	0.2 ~ 0.5 W/cm^2^	*E. coli, S. aureus, B. subtilis, P. aemginosa*	Cavitation cause bacteria damage.
28.5 kHz	0.5 W/cm^2^	*S. epidennidis, P. aeruginosa, E. coli*	After 48 hrs, no reduction of viable bacteria.
36 kHz	60 ~ 190 W/L	*Legionella pneumophila, Acanthamoeba castellanii*	US is able to use for biofilm removal, but not practical for large-scale application.
38 kHz	5 ~ 20 W/cm^2^	*E. coli*	US is able to use for water disinfection.
67 kHz	0.3 W/cm^2^	*E. coli, S. aureus, P. aemginosa*	Bacteria cells become susceptible to antibiotics through US treatment.
70 kHz	0.2 ~ 2 W/cm^2^	*E. coli*	Low frequency and intensity US increased the growth rate of the cells.
70 kHz	0.5 ~ 5 W/cm^2^	*P. aemginosa*	Low US frequency with high intensity has better removal efficiency than high frequency.
500 kHz	0.2 ~ 2 W/cm^2^	*E. coli*	High US frequency does not have high biocidal effect as low frequency with high intensity group.
800 kHz	5 ~ 20 W/cm^2^	*E. coli*	High US frequency leads to high biocidal effect. Chemical disinfectant activity can be improved as well.
850 kHz	0.2 ~ 2 W/cm^2^	*B. subtilis*	High US frequency leads to high biocidal effect.
